# Decay pattern of SARS-CoV-2 RNA surface contamination in real residences

**DOI:** 10.1038/s41598-024-54445-7

**Published:** 2024-03-14

**Authors:** Nan Lin, Bo Zhang, Rong Shi, Yu Gao, Zixia Wang, Zhiyi Ling, Ying Tian

**Affiliations:** 1 Department of Environmental Health, School of Public Health, Shanghai Jiao Tong University, 280 South Chongqing Rd, Shanghai, 200025 People’s Republic of China; 2 Huangpu Center for Disease Control and Prevention, 309 Xietu Rd, Shanghai, 200023 People’s Republic of China; 3 MOE-Shanghai Key Laboratory of Children’s Environmental Health, Xin Hua Hospital, Shanghai Jiao Tong University School of Medicine, Shanghai, 200092 People’s Republic of China

**Keywords:** SARS-CoV-2, Surface contamination, Fomite transmission, Decay, Residence, Environmental sciences, Health policy, Public health

## Abstract

The COVID-19 pandemic has provided valuable lessons that deserve deep thought to prepare for the future. The decay pattern of surface contamination by SARS-CoV-2 RNA in the residences of COVID-19 patients is important but still unknown. We collected 2,233 surface samples from 21 categories of objects in 141 residences of COVID-19 patients in Shanghai when attacked by the omicron variant in spring 2022. Several characteristics of the patients and their residences were investigated to identify relevant associations. The decay of contamination was explored to determine the persistence. Approximately 8.7% of the surface samples were tested positive for SARS-CoV-2 RNA. The basin, water tap, and sewer inlet had the highest positive rates, all exceeding 20%. Only time was significantly associated with the level of surface contamination with SARS-CoV-2, showing a negative association. The decrease fit a first-order decay model with a decay rate of 0.77 ± 0.07 day^−1^, suggesting a 90% reduction in three days. Positive associations between the cumulative number of newly diagnosed patients in the same building and the positive rate of SARS-CoV-2 RNA in the public corridor were significant during the three days. Our results, in conjunction with the likely lower infectivity or viability, demonstrate that fomite transmission played a limited role in COVID-19 spread. The time determined SARS-CoV-2 RNA contamination, which was reduced by three days. This study is the first to show the decay patterns of SARS-CoV-2 contamination in real residential environments, providing insight into the patterns of transmission, as well as community-based prevention and control of similar threats.

## Introduction

As of October 18, 2023, there were over 771 million COVID-19 cases, including over 6.97 million deaths worldwide^1^. As demonstrated by the World Health Organization (WHO), SARS-CoV-2 primarily spreads via droplet transmission and short-range airborne transmission when people are in close contact with each other and spreads in indoor settings via long-range airborne transmission^2,3^. An increasing body of evidence corroborates surface or fomite transmission^4,5,6^, which indicated that SARS-CoV-2 was transmitted between people who touched surfaces that a COVID-19 patient had just coughed or sneezed on or touched and then directly touched their mouth, nose, or eyes.

Viable SARS-CoV-2 is detectable on inanimate surfaces for up to 72 h^6^. Numerous studies have investigated SARS-CoV-2 contamination on object surfaces, but most have been performed in hospitals and healthcare settings^7–9^. Only a few studies have collected data from residences, of which the sample numbers were very limited^10–12^ and only one study collected a considerable number (N = 1232) of samples^13^. Therefore, surface contamination by SARS-CoV-2 in the residences of COVID-19 patients remains an unsolved issue for developing interventions to interrupt SARS-CoV-2 transmission, and information on the factors affecting surface contamination is very limited.

People spend over 80% of time indoors^14^, and even 100% of time in residential environment for quarantine during pandemic. Meanwhile, residential environment and community is one of the sites where COVID-19 infections are most likely to occur^15^ but also an understudied area. The SARS-CoV-2 omicron variant began to sweep the globe by the end of 2021^16^. Starting in March 2022, Shanghai, China, faced a severe wave of the pandemic caused by the omicron variant. From March 1 to May 31, 2022, Shanghai had a total of 626,806 COVID-19 cases (hereafter “patients”), including 591,341 asymptomatic carriers^17^, which rendered one of the severest hits by COVID-19 in China. A tiered regional approach to containment was implemented, based on the community. If the PCR test result was positive, patients were transported from their residences to hospitals for isolation, and all other residents in the same community were quarantined at their residences for 14 days. During quarantine, new patients emerged in the community, especially in old communities with high population densities, suggesting underlying potential community transmission. Unknown information about environmental contamination by SARS-CoV-2 in residences has aroused great doubts and concerns.

The present study characterized SARS-CoV-2 surface contamination of a wide range of objects in residences of COVID-19 patients in Shanghai during this wave of the pandemic and identified the potential determinants and decay patterns of the contamination. Persistence and potential fomite transmission were explored in conjunction with newly diagnosed patients in the community. Although the WHO has declared the end of the COVID-19 outbreak a global health emergency, we can still face the threat of a potentially infectious pandemic, both now and in the future. This study may provide clues to the patterns of transmission of similar threats, as well as to residents and policymakers of non-pharmaceutical interventions (NPIs) in the community.

## Materials and methods

### Sampling sites

Surface samples were collected from Huangpu District, Shanghai, which has a population of 24.87 million^18^. Huangpu District is an old downtown area in the city center with a population of 0.66 million and an area of 20.46 square kilometers, with a population density of 32,185 people per square kilometer, and it is the second highest density in Shanghai^19^. The area has both old and new residential buildings and communities, where residents have different living habits, making it a representative area to explore the surface contamination of SARS-CoV-2 under different conditions and the potential transmission in various communities. While, Shanghai is a super large city, and the representativeness of only one district is still limited.

Huangpu District has 59,280 cases, including 54,944 asymptomatic carriers, from March 1 to May 31, 2022, which was 9.5% of the cases in Shanghai^17^. The incidence rate of COVID-19 cases in Huangpu District was 9.0% (vs. 2.5% in Shanghai), rendering it the most severely affected district in Shanghai during this pandemic.

### Sample collection

During March 1–31, 2022 when the number of COVID-19 patients increase rapidly and stayed stable, we asked COVID-19 patients with positive results of the ORF1ab and N genes who lived in Huangpu District for permission for sample collection in their residences. A total of 188 patients responded, of which 8 were diagnosed between March 1 and 10, 98 between March 11 and 20, and 82 between March 21 and 31. The change in the number of confirmed cases showed that this month included both the beginning and the continuing phase of the severe wave of the pandemic. All participating patients officially provided informed consent for residence sampling and information collection. Twenty patients who were diagnosed as close contacts after transport were excluded, and 168 patients from 141 residences were included in the present study. The sites encompassed different types of residences, such as new apartments with elevators, and old communities with shared kitchens and bathrooms. Owing to the gradual increase in cases, most residences were sampled after mid-March. One to 17 residences were sampled on each sampling day, depending on the patient’s emergence and response. In each residence, samples from all available sites, that is, kitchens, bathrooms, bedrooms, living rooms, and public corridors, were collected. According to the previous studies, surface with higher contact frequency by people, e.g., handles, buttons, were especially included^7–9^. Each residence included 10 to 50 samples. A total of 2233 object surface samples from 21 categories were collected from five sites in almost all residences (Tables S1 and S2), which is substantially higher than the sample size of previous studies^10–13^, and ensured the richness of information and the reliability of data.

Sampling was performed according to the Health Industry Standard of China, WS/T 776-2021^20^. Briefly, after fully soaking the virus preservation solution (Shenqi Biotech, Shanghai, China) in the virus sampling tube, the sample swab (Shenqi Biotech, Shanghai, China) was smeared and rinsed repeatedly on the surface of the object more than three times. The entire surfaces of small objects, such as door handles, were sampled directly. Multipoint distributed sampling was performed for objects with large surfaces. Three to five areas were divided equally with at least 100 square centimeters for each area, and three to five areas on the object surface were sampled. After sampling, the handle of the sample swab was cut and discarded, and the swab was preserved in a sample tube containing virus preservation solution at 4 °C before testing. During sampling, at least one on-site blank sample and one transportation blank sample were used.

Patient information, including age, sex, first positive PCR results (ORF1ab gene Ct and N gene Ct value), COVID-19 vaccine, days after patient diagnosis (i.e., the time between the first positive test of the patient and the sampling of surfaces in residence), and days after patient transport (i.e., the time between the patient being transported from residence to designated hospitals and the sampling of surfaces in residence), was collected. The study protocol was approved by the Research Ethics Committee of the School of Public Health and Nursing at the Shanghai Jiao Tong University.

We also completed a walkthrough inspection of building and room features in residences, including residential areas, physical partitions (i.e., the presence of walls separating different functional sites or an open-space arrangement in residences), and the use of shared kitchens, shared bathrooms, and elevators (Table 1). Based on the effects of temperature and humidity on virus persistence^21^, we also collected meteorological information (Table S3) during the sampling period in March 2022 in Shanghai from http://data.cma.cn/.

**Table 1:** Population and residence characteristics.

Characteristic	Value^†^
**Population (N = 168)**	
Age (year)	49.8 ± 17.9 (51.0)
Gender	
Male	71 (42.3%)
Female	97 (57.7%)
ORF1ab gene Ct value	23.3 ± 5.9 (22.0)
N gene Ct value	23.5 ± 6.1 (23.0)
COVID-19 vaccine	
0	35 (20.8%)
1–2	68 (40.5%)
3	61 (36.3%)
Missing	4 (2.4%)
Days after patient diagnosis	2.8 ± 1.5 (2.0)
Days after patient transport	1.3 ± 1.2 (1.0)
**Residence (N = 141)**	
Residence area (m^2^)	87.1 ± 72.0 (70.0)
Physical partition	
Yes	122 (86.5%)
No	19 (13.5%)
Shared kitchen	
Yes	19 (13.5%)
No	122 (86.5%)
Shared bathroom	
Yes	21 (14.9%)
No	120 (85.1%)
Elevator	
Yes	45 (31.9%)
No	96 (68.1%)

^†^Values are expressed as mean ± SD (median) for age, ORF1ab gene Ct value, N gene Ct value, days after patient diagnosis, days after patient transport, and residence area, and as numbers and percentages for other characteristics.

### RNA extraction and RT-qPCR

A SARS-CoV-2 Nucleic Acid Test Kit (Beijing Applied Biological Technologies, Beijing, China) was used to extract RNA^22,23^. The sample to be tested was heated at 56 °C for 30 min to inactivate the virus. A 200-μL sample was placed in a tube, and 10 μL of an internal standard was added. Next, 5 μL of the mixture was added to the PCR system. The PCR system consisted of nuclease-free water, nucleic acid amplification reaction solution, 20 × reverse transcriptase and a 10 × O/N reaction solution of primers and probes (Beijing Applied Biological Technologies, Beijing, China). After mixing, the 15 μL mixture was transferred to a PCR tube for ORF1ab and N gene amplification using a Quant Studio™ 7 Flex Real-Time PCR System (Thermo Fisher Scientific, Waltham, USA). Both ORF1ab and N targets are highly conserved (i.e., less likely to mutate) and highly-specific for confirming SARS-CoV-2^24^. The cycling protocol was 45 °C for 10 min (1 cycle); 95 °C for 5 min (1 cycle); then 95 °C for 15 s, and 60 °C for 45 s (total 45 cycles). The target sequences of open reading frame 1ab (ORF1ab) and nucleocapsid protein (N) are listed below:

1. ORF1ab
  F: CCCTGTGGGTTTTACACTTAA.
  R: ACGATTGTGCATCAGCTGA.
  P: 5′-FAM-CCGTCTGCGGTATGTGGAAAGGTTATGG-BHQ1-3′.
2. N
  F: GGGGAACTTCTCCTGCTAGAAT.
  R: CAGACATTTTGCTCTCAAGCTG.
  P: 5′-FAM-TTGCTGCTGCTTGACAGATT-TAMRA-3′.

Positive and negative results of the samples were determined according to the manufacturer’s instructions. (1) If Ct ≤ 38 and an S-type amplification curve were generated, ORF1ab and/or N genes were identified as positive. (2) If 38 < Ct < 40, the samples were re-examined, and if Ct was < 40 and there was an S-type amplification curve, the result was identified as positive. If Ct was ≥ 40, the result was considered negative. (3) If Ct ≥ was 40, the target gene was identified as negative. If the ORF1ab and N genes were positive, the surface sample was considered positive for SARS-CoV-2 RNA^23^. We used the positivity rates of SARS-CoV-2 RNA (PR_CoV_) to identify surface contamination.

Each batch included 50 samples. Four acid-positive control (recombinant vector containing ORF1ab and N gene fragments) and three negative control (one blank, one physiological saline and one negative control from test kit) were also examined for each batch. All on-site and transportation blank samples tested negative. Ct values of acid-positive controls were 30, and both the intra-day and inter-day coefficients of variation were lower than 5%.

### Data analysis

Values are expressed as mean ± standard deviation (SD) and median (in brackets) for age, ORF1ab gene Ct value, N gene Ct value, days after patient diagnosis, days after patient transport, and residence area, and as numbers and percentages for other population and residence characteristics.

The Mann–Whitney *U* test was performed for PR_CoV_ comparisons between two sample groups, including gender, physical partition, and the use of shared kitchens, shared bathrooms, and elevators. Spearman correlation analysis was performed for the association of PR_CoV_ with age, ORF1ab gene Ct value, N gene Ct value, residence area, COVID-19 vaccine, days after patient diagnosis, and days after patient transport, and for the association between PR_CoV_ in the public corridors of buildings and newly diagnosed patients in buildings.

Linear regression models [Eq. (1)] and first-order decay [Eq. (2)]^25–27^ were simulated to fit the decrease in PR_CoV_ in residential areas.

1 $$ {\text{PR}}_{{\text{t}}} = {\text{ PR}}_{0} {-}{\text{ K}} \times {\text{T}} $$

2 $$ {\text{PR}}_{{\text{t}}} = {\text{ PR}}_{0} \times {\text{e}}^{{ - {\text{ K}} \times {\text{T}}}} $$

where PR_t_ is the positive rate at time t, PR_0_ is the original positive rate, K (day^−1^) is the decay rate, and T (days) is time. Both days after patient diagnosis and days after patient transport were used as time to fit the simulation.

Based on these models, the number of days required to achieve a 90% reduction was estimated using Eqs. (3 and 4).

3 $$ {\text{T}}_{{{9}0}} = \, 0.{9}/{\text{K}} $$

4 $$ {\text{T}}_{{{9}0}} = \, {-}{\text{ ln }}\left( {0.{1}} \right)/{\text{K}} $$

Analyses were performed using SPSS (SPSS, Inc., Chicago, Illinois, USA) and R 4.2.0 (R Foundation, Vienna, Austria). All statistical tests were two-sided with a type-I error rate of 0.05.

## Results and discussion

### Characteristics in population and residence

The characteristics of the 168 COVID-19 patients and their 141 living residences are summarized in Table 1. Most of the patients were middle-aged. The Ct values of the patients indicated a relatively high viral load. Approximately 80% of the patients were vaccinated. The samples of the object surface were primarily collected three days after patient diagnosis (first positive PCR test), which was generally one day after patient transport. The mean and median areas of residential environments were 87.1 and 70.0 m^2^, respectively, generally smaller than the national average of 111 square meters^18^. Approximately 14% of the residences had no physical partitions (i.e., 14% of the residences had an open space arrangement). Similar proportions (14% and 15%, respectively) of residents used shared kitchens and bathrooms. One-third (32%) of the residences were equipped with elevators.

### Positive rate of SARS-CoV-2 RNA on object surfaces in residences

Several previous studies have substantiated the disparity in the results of SARS-CoV-2 RNA measurement methods^28^. The present study used RT-qPCR, which has high sensitivity for nucleic acid amplification and potential false-positive results^29^. Despite the wide detection of SARS-CoV-2 RNA on surfaces, viable viruses have not been confirmed in any positive RNA samples in previous studies^30–32^. Although transmission risks require further validation, our results provide several clues regarding contamination.

**Figure 1:**
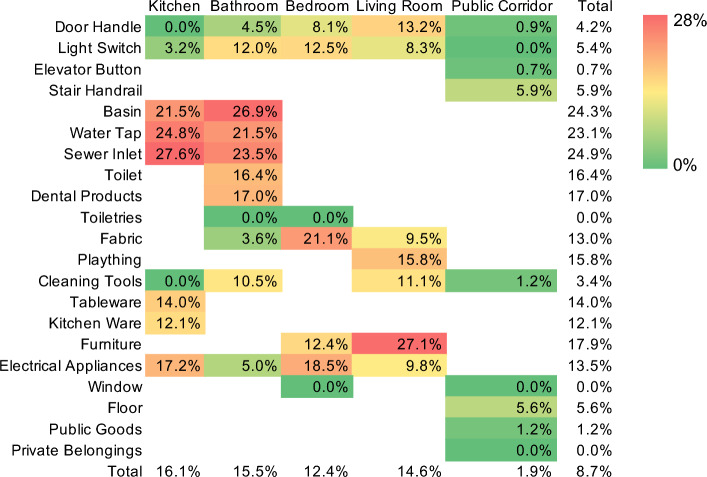
Heatmap of positive rates of SARS-CoV-2 (PR_CoV_) aggregation by setting in residences.

The PR_CoV_ of the total aggregated object surface samples in residences in the present study was 8.7% (Fig. 1), which was lower than that of hospitals and quarantine rooms in previous studies^33–35^, but similar to a community study (8.3%)^36^, suggesting little contamination with SARS-CoV-2 in the patients’ residences. A significantly higher PR_CoV_ was found in the residences of multiple patients than in those of a single patient (*p* < 0.05, Fig. S1), except for the public corridor in buildings, which suggests that multiple COVID-19 patients greatly exacerbate cross-contamination in the environment. Previous studies have reported no significant difference of PR_CoV_ with COVID-19 in hospitals and isolation units^30^.

Basins, water taps, and sewer inlets had the highest PR_CoV_ at 24, 23, and 25%, respectively (Fig. 1). Unclean hands are the culprit of infection via fomite transmission^37,38^. The high PR_CoV_ of water taps and related objects in the present study may be a result of a high frequency of contamination by unclean hands, water, and water droplets. This result suggests that hand washing is an effective method to clean hands, but it is also a reminder of the need for extra disinfection of washing areas. People should be aware of re-contamination by touching water taps after hand washing, especially in communities with shared kitchens and bathrooms, which enhances the potential for surface (fomite) transmission^39^. Several reports have found high contamination in sewer inlets in drainage systems^40^. Even in the absence of gastrointestinal symptoms, SARS-CoV-2 RNA has been detected in fecal samples from over 40% of patients^41^.

Inside the patients’ residences, PR_CoV_ ranged from 12 to 16% at different sites, without significant differences. Basins, water taps, and sewer inlets had the highest PR_CoV_ in the kitchen and bathroom. Fabric was a contributor to the highest PR_CoV_ in bedrooms, which was primarily bedding (Table S1), consistent with the findings of previous studies^12,34,42^. Furniture in living rooms had a high PR_CoV_ of 27%, but the total PR_CoV_ in furniture was 18%. The public corridor had the lowest PR_CoV_ at 2% (Fig. 1), suggesting minimal risk via fomite transmission in the community. Stair handrails and floors had the highest PR_CoV_ (6%) in the public corridors.

### Potentially relevant characteristics of patient and residence

According to the influence of patient number on surface contamination by SARS-CoV-2, residences with multiple patients were excluded for further analyses and discussion. Basins, water taps, and sewer inlets were integrated as washing basins for further analysis based on their high PR_CoV_. The associations between PR_CoV_ and different characteristics of patients and residences in single-patient residences are summarized in Table 2. As the number of days after the patient was transported increased, PR_CoV_ in the residence decreased significantly, particularly in the bathroom and washing basin. An increase in days after patient diagnosis was also significantly associated with decreasing PR_CoV_ in residences, but not at any particular site, except in the washing basin. No other factors were significantly associated with PR_CoV_ infection in residential areas. Gender, physical partition, and the use of a shared kitchen, shared bathroom, and elevator did not play a role in the PR_CoV_ difference. However, a previous study demonstrated a decrease in surface contamination with increasing distance from the patient^8^.

**Table 2:** Correlation coefficients between positive rates of SARS-CoV-2 (PR_CoV_) and characteristics of patients and residences in single-patient residences.

Characteristic	Correlation coefficients between PR_CoV_ and characteristics						
	Residence	Kitchen	Bathroom	Bedroom	Living room	Public corridor in building	Washing basin
Age	− 0.02	0.02	0.23	0.22	0.20	− 0.0004	0.11
ORF1ab gene Ct value	− 0.05	− 0.14	− 0.15	− 0.09	0.04	− 0.10	− 0.10
N gene Ct value	− 0.02	− 0.12	− 0.15	− 0.07	0.05	− 0.10	− 0.08
COVID-19 vaccine	0.03	0.01	− 0.17	− 0.11	0.11	0.01	0.003
Days after patient diagnosis	− 0.27**	− 0.19	− 0.12	− 0.12	− 0.01	− 0.04	− 0.23
Days after patient transport	− 0.33***	− 0.13	− 0.31*	− 0.03	− 0.02	− 0.18*	− 0.36**
Residence area	− 0.05	− 0.02	− 0.04	0.04	− 0.004	− 0.10	− 0.004

*p < 0.05, **p < 0.01, ***p < 0.001.

Transporting COVID-19 patients from their residences to designated hospitals was one of the NPIs in China. This measure was beneficial for controlling disease progression in patients. The present study showed its effectiveness in reducing surface contamination and potential community transmission. With the loosening of epidemic prevention policies, conscious home isolation of COVID-19 patients is dominant globally. While, the results of the present study imply a potential surface contamination problem. Especially in old communities, the residence environment, for example, the use of shared kitchens and bathrooms, may not provide conditions for home isolation to COVID-19 patients and may enhance cross-contamination in these communities. These findings provide clues for low- and middle-income countries regarding community-based prevention and control of similar infectious disease threats in communities with high population densities such as slums^43^.

### Decay of positive rate

**Figure 2:**
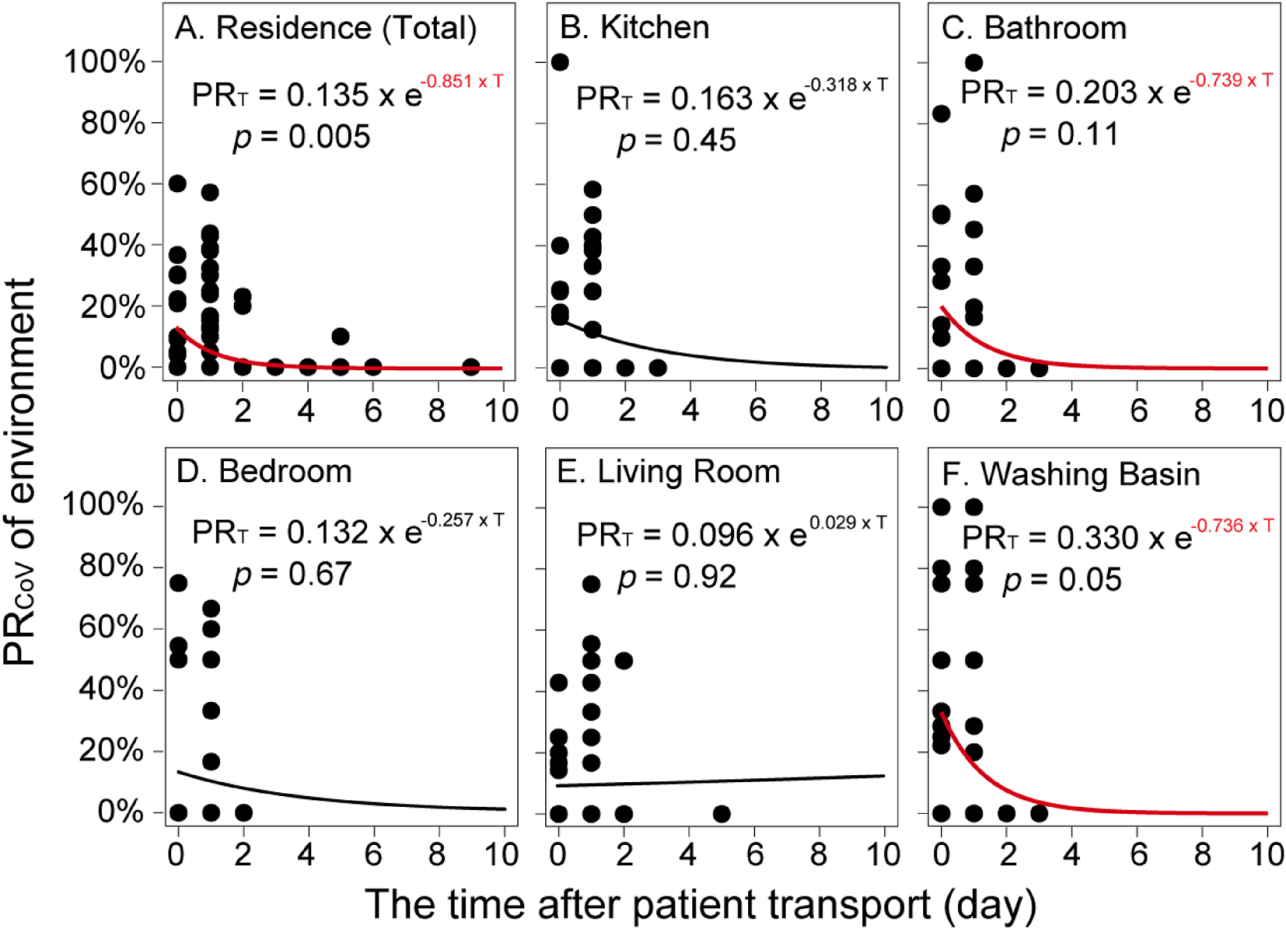
First-order decay models (formulas in figure) of positive rates of SARS-CoV-2 (PR_CoV_) in the residence environment over time after patient transport.

Based on the aforementioned findings, we further analyzed the decrease in PR_CoV_ with increasing days after the patient transport. The fitting formulas used the linear regression model and first-order decay model. Three days after patient transport, PR_CoV_ decreased to 0% at all sites (Fig. 2). Linear regression models did not identify a stable decay rate K (Table S4), but first-order decay models found a relatively consistent result of decay rate K as 0.77 ± 0.07 day^−1^ for PR_CoV_ at several sites (Fig. 2), including residences (K = 0.85 day^−1^), bathrooms (K = 0.74 day^−1^), and washing basins (K = 0.74 day^−1^). Therefore, the first-order decay model was fitted using PR_t_ = PR_0_ × e ^(− 0.77±0.07) × T^, which indicated a 90% reduction of PR_CoV_ in 3.0 ± 0.3 days, i.e., T_90_ = − ln (0.1)/(− 0.77 ± 0.07) = 3.0 ± 0.3 days. Laboratory studies have revealed a 99% reduction in infectious SARS-CoV-2 on object surfaces generally within three days in indoor environmental conditions^6,44,45^. Although laboratory studies have always optimized the recovery of viruses from surfaces by simulating the worst scenario, our results substantiate the reduction findings. Several previous studies also estimated a 90% reduction of SARS-CoV-2 in 2.2–3.8 days in river water and seawater at different temperatures^27^, 1.6–2.1 days in wastewater and 2.0 days in tap water at room temperature^46^, which are similar to the findings in the present study, including the temperature (Table S3). Our study is the first to present the same rules for surfaces in real residential environments.

We observed a significant positive association between the cumulative number of newly diagnosed patients in the same building from the day when the patient was transported and PR_CoV_ in the public corridor in the building, and the significance only occurred in the first three days after the patient was transported (Table S5). This time period of three days is consistent with the decay findings above. This may serve as an evidence that surface contamination in patients’ residences has a potential transmission risk only in the first three days if the contamination source is gone. However, it was difficult to determine whether the increase in surface contamination was caused by an increasing number of patients or whether the potential transmission induced by surface contamination led to an increasing number of patients in residential buildings. For example, the positivity rate of surfaces was associated with COVID-19 disease dynamics in a longitudinal community study, and the authors proposed an early warning monitoring tool for the environmental surveillance of surfaces to inform disease dynamics^36^. We cannot exclude the effects of daily COVID-19 testing for all residents in the community on early screening after the first patient was diagnosed. Therefore, this result requires further investigation.

Notably, the positive associations remained significant even when disinfection was performed in residences immediately after sampling, which suggests that disinfection plays a limited role in reducing contamination. As validated by previous studies, hand hygiene plays a major role in reducing fomite transmission. When and how often surface disinfection is performed has little impact on reducing estimated risks^36,38,5^. Environmental disinfection in residences needs more consideration, especially concerning increased exposure to cleaners and disinfectants^47,48^.

### Study strengths and limitations

Our study has several strengths. A wide range of surface samples from a substantial number of residences was collected to characterize SARS-CoV-2 contamination, portraying a complete picture of surface contamination in patients’ residences. In addition to the potentially relevant factors investigated in other studies, this study identified a reduction period of three days using a decay model. To our knowledge, this is the first study to reveal the decay pattern of SARS-CoV-2 RNA contamination on surfaces in real living environments. Multiple associations between surface contamination and the number of diagnosed cases, such as the positive rate in the public corridor and its association with newly diagnosed patients in buildings, were investigated to discuss potential fomite transmission. This study had some limitations. Our results do not directly indicate transmission risks because only SARS-CoV-2 RNA levels were measured. The real-time temperature and relative humidity were not monitored in the field. The finding of contamination reduction over time was based on cross-sectional sampling, which requires further verification in longitudinal studies.

## Conclusions

The present study used a wide range of surface samples to characterize SARS-CoV-2 RNA contamination in patients’ residences. The environmental contamination of object surfaces by SARS-CoV-2 RNA in residences of COVID-19 patients is low, but cross-contamination caused by water taps and washing basins may have hidden transmission risks. Generally, fomite transmission played a limited role in COVID-19 spread. Even the residence was contaminated by SARS-CoV-2, it was reduced by 90% three days after the patient left. This reduction suggests that transporting patients from residences may be an effective nonpharmaceutical intervention to impede environmental contamination. This provides clues for community-based prevention and control of COVID-19, especially in communities with a high population density.

### Supplementary Information

Supplementary Information.

## Data Availability

The datasets generated and/or analyzed during the current study are not publicly available due to the confidentiality of participants’ personal information required by the Research Ethics Committee but are partly available from the corresponding author on reasonable request.

## References

[1] WHO. *WHO Coronavirus (COVID-19) Dashboard*. (2023). https://covid19.who.int/. Accessed 18 Oct 2023.

[2] WHO. *Coronavirus Disease (COVID-19): How is it Transmitted?* (2021). https://www.who.int/emergencies/diseases/novel-coronavirus-2019/question-and-answers-hub/q-a-detail/coronavirus-disease-covid-19-how-is-it-transmitted. Accessed 9 Jun 2022.

[3] Yao M (2022). SARS-CoV-2 aerosol transmission and detection. Eco-Environ. Health.

[4] Edward DGF (1941). Resistance of influenza virus to drying and its demonstration on dust. Lancet.

[5] US CDC. *Science Brief: SARS-CoV-2 and Surface (Fomite) Transmission for Indoor Community Environments*. (2021). https://www.cdc.gov/coronavirus/2019-ncov/more/science-and-research/surfacetransmission.html. Accessed 9 Jun 2022. 34009771

[6] van Doremalen N, Bushmaker T, Morris DH, Holbrook MG, Gamble A, Williamson BN, Tamin A, Harcourt JL, Thornburg NJ, Gerber SI, Lloyd-Smith JO, de Wit E, Munster VJ (2020). Aerosol and surface stability of SARS-CoV-2 as compared with SARS-CoV-1. N. Engl. J. Med..

[7] Chia PY, Coleman KK, Tan YK, Ong SWX, Gum M, Lau SK, Lim XF, Lim AS, Sutjipto S, Lee PH, Son TT, Young BE, Milton DK, Gray GC, Schuster S, Barkham T, De PP, Vasoo S, Chan M, Ang BSP, Tan BH, Leo YS, Ng OT, Wong MSY, Marimuthu K, Singapore Novel Coronavirus Outbreak Research (2020). Detection of air and surface contamination by SARS-CoV-2 in hospital rooms of infected patients. Nat. Commun..

[8] Razzini K, Castrica M, Menchetti L, Maggi L, Negroni L, Orfeo NV, Pizzoccheri A, Stocco M, Muttini S, Balzaretti CM (2020). SARS-CoV-2 RNA detection in the air and on surfaces in the COVID-19 ward of a hospital in Milan, Italy. Sci. Total Environ..

[9] Wu S, Wang Y, Jin X, Tian J, Liu J, Mao Y (2020). Environmental contamination by SARS-CoV-2 in a designated hospital for coronavirus disease 2019. Am. J. Infect. Control.

[10] Correia G, Rodrigues L, Afonso M, Mota M, Oliveira J, Soares R, Tomas AL, Reichel A, Silva PM, Costa JJ, da Silva MG, Santos NC, Goncalves T (2022). SARS-CoV-2 air and surface contamination in residential settings. Sci. Rep..

[11] Hu X, Xing Y, Ni W, Zhang F, Lu S, Wang Z, Gao R, Jiang F (2020). Environmental contamination by SARS-CoV-2 of an imported case during incubation period. Sci. Total Environ..

[12] Marcenac P, Park GW, Duca LM, Lewis NM, Dietrich EA, Barclay L, Tamin A, Harcourt JL, Thornburg NJ, Rispens J, Matanock A, Kiphibane T, Christensen K, Pawloski LC, Fry AM, Hall AJ, Tate JE, Vinje J, Kirking HL, Pevzner E (2021). Detection of SARS-CoV-2 on surfaces in households of persons with COVID-19. Int. J. Environ. Res. Public Health.

[13] Shragai T, Pratt C, CastroGeorgi J, Donnelly MAP, Schwartz NG, Soto R, Chuey M, Chu VT, Marcenac P, Park GW, Ahmad A, Albanese B, Totten SE, Austin B, Bunkley P, Cherney B, Dietrich EA, Figueroa E, Folster JM, Godino C, Herzegh O, Lindell K, Relja B, Sheldon SW, Tong S, Vinje J, Thornburg NJ, Matanock AM, Hughes LJ, Stringer G, Hudziec M, Beatty ME, Tate JE, Kirking HL, Hsu CH, Team C-HT (2021). Household characteristics associated with surface contamination of SARS-CoV-2 and frequency of RT-PCR and viral culture positivity-California and Colorado, 2021. PLoS ONE.

[14] Ministry of Environmental Protection the People’s Republic of China (2013). Exposure Factors Handbook of Chinese Population (Adults).

[15] Akaishi T, Kushimoto S, Katori Y, Kure S, Igarashi K, Takayama S, Abe M, Tanaka J, Kikuchi A, Onodera K, Ishii T (2021). COVID-19 transmission in group living environments and households. Sci. Rep..

[16] WHO. *Update on Omicron*. (2021). https://www.who.int/news/item/28-11-2021-update-on-omicron. Accessed 9 Jun 2022.

[17] Shanghai Municipal Health Commission. *Daily information of confirmed cases and asymptomatic carriers in each district*. (2022). https://wsjkw.sh.gov.cn/index.html. Accessed 9 Jun 2022.

[18] China National Bureau of Statistics. *China Population Census Yearbook 2020*. (2022). http://www.stats.gov.cn/tjsj/pcsj/rkpc/7rp/indexch.htm. Accessed 7 Jul 2022.

[19] Shanghai Bureau of Statistics. *Shanghai Statistical Yearbook*. (2022). https://tjj.sh.gov.cn/tjnj/index.html. Accessed 15 Jun 2022.

[20] National Health Commission of P.R. China. *Specification for Environmental Monitoring of SARS-CoV-2 in Agriculture Product Markets and Trade Markets (WS/T 776–2021)*. (2021).

[21] Wei Y, Dong Z, Fan W, Xu K, Tang S, Wang Y, Wu F (2022). A narrative review on the role of temperature and humidity in COVID-19: Transmission, persistence, and epidemiological evidence. Eco-Environ. Health.

[22] Chang D, Chen Z, Wang FS, Xie LX, Dela Cruz CS, Sharma L, Qin EQ (2020). Host tolerance contributes to persistent viral shedding in COVID-19. Eclin. Med..

[23] Guo JJ, Yu YH, Ma XY, Liu YN, Fang Q, Qu P, Guo J, Lou JL, Wang YJ (2020). A multiple-center clinical evaluation of a new real-time reverse transcriptase PCR diagnostic kit for SARS-CoV-2. Future Virol..

[24] Tran NK, Miller C, Waldman S (2021). The SARS-CoV-2 Variant and its Impact on Diagnostic Testing.

[25] Chick H (1908). An investigation of the laws of disinfection. J. Hyg..

[26] Nastasi N, Renninger N, Bope A, Cochran SJ, Greaves J, Haines SR, Balasubrahmaniam N, Stuart K, Panescu J, Bibby K, Hull NM, Dannemiller KC (2022). Persistence of viable MS2 and Phi6 bacteriophages on carpet and dust. Indoor Air.

[27] Sala-Comorera L, Reynolds LJ, Martin NA, O'Sullivan JJ, Meijer WG, Fletcher NF (2021). Decay of infectious SARS-CoV-2 and surrogates in aquatic environments. Water Res..

[28] Lv J, Yang J, Xue J, Zhu P, Liu L, Li S (2020). Detection of SARS-CoV-2 RNA residue on object surfaces in nucleic acid testing laboratory using droplet digital PCR. Sci. Total Environ..

[29] Braunstein GD, Schwartz L, Hymel P, Fielding J (2021). False positive results with SARS-CoV-2 RT-PCR tests and how to evaluate a RT-PCR-positive test for the possibility of a false positive result. J. Occup. Environ. Med..

[30] Goncalves J, da Silva PG, Reis L, Nascimento MSJ, Koritnik T, Paragi M, Mesquita JR (2021). Surface contamination with SARS-CoV-2: A systematic review. Sci. Total Environ..

[31] Moreno T, Pinto RM, Bosch A, Moreno N, Alastuey A, Minguillon MC, Anfruns-Estrada E, Guix S, Fuentes C, Buonanno G, Stabile L, Morawska L, Querol X (2021). Tracing surface and airborne SARS-CoV-2 RNA inside public buses and subway trains. Environ. Int..

[32] Santarpia JL, Rivera DN, Herrera VL, Morwitzer MJ, Creager HM, Santarpia GW, Crown KK, Brett-Major DM, Schnaubelt ER, Broadhurst MJ, Lawler JV, Reid SP, Lowe JJ (2020). Aerosol and surface contamination of SARS-CoV-2 observed in quarantine and isolation care. Sci. Rep..

[33] Dargahi A, Jeddi F, Vosoughi M, Karami C, Hadisi A, Ahamad Mokhtari S, Ghobadi H, Alighadri M, Haghighi SB, Sadeghi H (2021). Investigation of SARS CoV-2 virus in environmental surface. Environ. Res..

[34] Hu X, Ni W, Wang Z, Ma G, Pan B, Dong L, Gao R, Jiang F (2021). The distribution of SARS-CoV-2 contamination on the environmental surfaces during incubation period of COVID-19 patients. Ecotoxicol. Environ. Saf..

[35] Ye G, Lin H, Chen S, Wang S, Zeng Z, Wang W, Zhang S, Rebmann T, Li Y, Pan Z, Yang Z, Wang Y, Wang F, Qian Z, Wang X (2020). Environmental contamination of SARS-CoV-2 in healthcare premises. J. Infect..

[36] Harvey AP, Fuhrmeister ER, Cantrell ME, Pitol AK, Swarthout JM, Powers JE, Nadimpalli ML, Julian TR, Pickering AJ (2021). Longitudinal monitoring of SARS-CoV-2 RNA on high-touch surfaces in a community setting. Environ. Sci. Technol. Lett..

[37] Pitol AK, Bischel HN, Kohn T, Julian TR (2017). Virus transfer at the skin-liquid interface. Environ. Sci. Technol..

[38] Pitol AK, Julian TR (2021). Community transmission of SARS-CoV-2 by surfaces: Risks and risk reduction strategies. Environ. Sci. Technol. Lett..

[39] Stoler J, Jepson WE, Wutich A (2020). Beyond handwashing: Water insecurity undermines COVID-19 response in developing areas. J. Glob. Health.

[40] Feng B, Xu K, Gu S, Zheng S, Zou Q, Xu Y, Yu L, Lou F, Yu F, Jin T, Li Y, Sheng J, Yen HL, Zhong Z, Wei J, Chen Y (2021). Multi-route transmission potential of SARS-CoV-2 in healthcare facilities. J. Hazard. Mater..

[41] Wong MC, Huang J, Lai C, Ng R, Chan FKL, Chan PKS (2020). Detection of SARS-CoV-2 RNA in fecal specimens of patients with confirmed COVID-19: A meta-analysis. J. Infect..

[42] Wei L, Huang W, Lu X, Wang Y, Cheng L, Deng R, Long H, Zong Z (2020). Contamination of SARS-CoV-2 in patient surroundings and on personal protective equipment in a non-ICU isolation ward for COVID-19 patients with prolonged PCR positive status. Antimicrob. Resist. Infect. Control.

[43] Ding Z, Xie L, Guan A, Huang D, Mao Z, Liang X (2020). Global COVID-19: Warnings and suggestions based on experience of China. J. Glob. Health.

[44] Biryukov J, Boydston JA, Dunning RA, Yeager JJ, Wood S, Reese AL, Ferris A, Miller D, Weaver W, Zeitouni NE, Phillips A, Freeburger D, Hooper I, Ratnesar-Shumate S, Yolitz J, Krause M, Williams G, Dawson DG, Herzog A, Dabisch P, Wahl V, Hevey MC, Altamura LA (2020). Increasing temperature and relative humidity accelerates inactivation of SARS-CoV-2 on surfaces. MSphere.

[45] Chin AWH, Chu JTS, Perera MRA, Hui KPY, Yen HL, Chan MCW, Peiris M, Poon LLM (2020). Stability of SARS-CoV-2 in different environmental conditions. Lancet Microb..

[46] Bivins A, Greaves J, Fischer R, Yinda KC, Ahmed W, Kitajima M, Munster VJ, Bibby K (2020). Persistence of SARS-CoV-2 in water and wastewater. Environ. Sci. Technol. Lett..

[47] Chang A, Schnall AH, Law R, Bronstein AC, Marraffa JM, Spiller HA, Hays HL, Funk AR, Mercurio-Zappala M, Calello DP, Aleguas A, Borys DJ, Boehmer T, Svendsen E (2020). Cleaning and disinfectant chemical exposures and temporal associations with COVID-19: National poison data system, United States, January 1, 2020-March 31, 2020. MMWR Morb. Mort. Wkly. Rep..

[48] Zheng G, Filippelli GM, Salamova A (2020). Increased indoor exposure to commonly used disinfectants during the COVID-19 pandemic. Environ. Sci. Technol. Lett..

