# Supplementary material for: Decay pattern of SARS-CoV-2 RNA surface contamination in real residences
Source: Sci Rep. 2024 Mar 14;14:6190. doi: 10.1038/s41598-024-54445-7 (PMC10940586; doi:10.1038/s41598-024-54445-7)
Supplement: Supplementary file 1 — Supplementary Information. [file 41598_2024_54445_MOESM1_ESM.docx]

**Supporting Information**

**Decay pattern of SARS-CoV-2 RNA surface contamination in real residences**

Nan Lin^ϯ,1^, Bo Zhang^ǂ,1^, Rong Shi^ϯ,1^, Yu Gao^ϯ^, Zixia Wang^ϯ^, Zhiyi Ling^ǂ,^*, Ying Tian^ϯ,₤,^*

^ϯ^ Department of Environmental Health, School of Public Health, Shanghai Jiao Tong University, Shanghai 200025, China

^ǂ^ Huangpu Centers for Disease Control and Prevention, Shanghai 200023, P. R. China

^₤^ MOE-Shanghai Key Laboratory of Children's Environmental Health, Xin Hua Hospital, Shanghai Jiao Tong University School of Medicine, Shanghai, China

^1^ These authors contributed equally.

*Corresponding authors:

**Ying Tian, MD**

Professor, Department of Environmental Health

School of Public Health, Shanghai Jiao Tong University

280 South Chongqing Rd, Shanghai 200025, China

Tel: +86-021-64663944 Email: tianmiejp@sjtu.edu.cn

**Zhiyi Ling**

Huangpu Centers for Disease Control and Prevention

309 Xietu Rd, Shanghai 200023, China

Tel: +86-13701983602 Email: [lingzhiyi002@126.com](mailto:lingzhiyi002@126.com)

**This file includes:**

Table S1 Category and site of different objectives studied.

Table S2 Sampling numbers of different studied objectives.

Table S3 Meteorological information during the sampling period in March 2022 in Shanghai.

Table S4 Estimated decay models of positive rates of SARS-CoV-2 in the residence environment.

Table S5 Correlation coefficients between positive rates of SARS-CoV-2 in public corridors and the cumulative numbers of newly diagnosed patients in the same building from the day of patient transport.

Figure S1 Heatmaps of positive rates of SARS-CoV-2 in residences of single and multiple patients.

Table S1 Category and site of different objectives studied.

| **Category/Site** | **Kitchen** | **Bathroom** | **Bedroom** | **Living room** | **Public corridor** |
| --- | --- | --- | --- | --- | --- |
| **Door handle** | Door handle of kitchen | Door handle of bathroom | Door handle of bedroom | Door handle of house | Door handle of building gate |
| **Light switch** | Light switch in kitchen | Light switch in bathroom | Light switch in bedroom | Light switch in living room | Light switch in corridor |
| **Elevator button** | / | / | / | / | Elevator button in elevator |
| **Stair handrail** | / | / | / | / | Stair handrail in building |
| **Basin** | Internal surface of basin in kitchen | Internal surface of basin in bathroom | / | / | / |
| **Water tap** | Water tap in kitchen | Water tap in bathroom | / | / | / |
| **Sewer inlet** | Sewer inlet in kitchen | Sewer inlet in bathroom | / | / | / |
| **Toilet** | / | Sit toilet or squat toilet | / | / | / |
| **Dental products** | / | Toothbrush and tooth cup | / | / | / |
| **Toiletries** | / | Shampoo | Skin care products | / | / |
| **Fabric** | / | Towel | Quilt, blanket, and bed sheet | Sofa and carpet | / |
| **Plaything** | / | / | / | Toy, stationery, and pet supplies | / |
| **Cleaning tools** | Trash can, broom, and mop | Trash can | / | Trash can | Public trash can |
| **Tableware** | Chopsticks, spoon, and drinking cup | / | / | / | / |
| **Kitchen ware** | Cutting board, kitchen knife and knife rest | / | / | / | / |
| **Furniture** | / | / | Closet | Table, chair, and closet | / |
| **Electrical appliances** | Refrigerator, microwave, and electric kettle | Washing machine, dryer, and remote control of toilet | TV | Table lamp | / |
| **Window** | / | / | Window in bedroom | / | Window in building |
| **Floor** | / | / | / | / | Floor at the door |
| **Public goods** | / | / | / | / | Mail box, milk box, water meter box, and shelf |
| **Private belongings** | / | / | / | / | Shoebox, and private closet |

Table S2 Sampling numbers of different studied objectives.

| **Category/Site** | **Kitchen** | **Bathroom** | **Bedroom** | **Living room** | **Public corridor** | **Total** |
| --- | --- | --- | --- | --- | --- | --- |
| **Door handle** | 12 | 45 | 37 | 76 | 234 | 404 |
| **Light switch** | 16 | 25 | 20 | 48 | 77 | 186 |
| **Elevator button** | 0 | 0 | 0 | 0 | 147 | 147 |
| **Stair handrail** | 0 | 0 | 0 | 0 | 188 | 188 |
| **Basin** | 12 | 13 | 0 | 0 | 0 | 25 |
| **Water tap** | 33 | 36 | 0 | 0 | 0 | 69 |
| **Sewer inlet** | 37 | 67 | 0 | 0 | 0 | 104 |
| **Toilet** | 0 | 70 | 0 | 0 | 0 | 70 |
| **Dental products** | 0 | 26 | 0 | 0 | 0 | 26 |
| **Toiletries** | 0 | 1 | 3 | 0 | 0 | 4 |
| **Fabric** | 0 | 8 | 19 | 21 | 0 | 48 |
| **Plaything** | 0 | 0 | 0 | 19 | 0 | 19 |
| **Cleaning tools** | 6 | 19 | 0 | 9 | 83 | 117 |
| **Tableware** | 22 | 0 | 0 | 0 | 0 | 22 |
| **Kitchen ware** | 83 | 0 | 0 | 0 | 0 | 83 |
| **Furniture** | 0 | 0 | 116 | 68 | 0 | 184 |
| **Electrical appliances** | 99 | 20 | 14 | 72 | 0 | 205 |
| **Window** | 0 | 0 | 4 | 0 | 17 | 21 |
| **Floor** | 0 | 0 | 0 | 0 | 36 | 36 |
| **Public goods** | 0 | 0 | 0 | 0 | 242 | 242 |
| **Private belongings** | 0 | 0 | 0 | 0 | 33 | 33 |
| **Total** | 320 | 330 | 213 | 313 | 1057 | 2233 |

Table S3 Meteorological information during the sampling period in March 2022 in Shanghai.

| **Meteorological factor** | **Mean ± SD** | **Median** | **Min** | **Max** |
| --- | --- | --- | --- | --- |
| Maximum temperature (°C) | 17 ± 5 | 18 | 10 | 26 |
| Minimum temperature (°C) | 10 ± 3 | 9 | 5 | 17 |
| Relative humidity (%) | 67 ± 19 | 61 | 36 | 100 |

Table S4 Estimated decay models of positive rates of SARS-CoV-2 (PR_CoV_) in the residence environment.

| **PR_t_ = PR_0_ × e^– K × T^** | **T = days after patient diagnosed** | | |  | **T = days after patient transported** | | |
| --- | --- | --- | --- | --- | --- | --- | --- |
|  | **PR_0_** | **K** | ***p for K*** |  | **PR_0_** | **K** | ***p for K*** |
| Residence | 0.22 | 0.54 | 0.005 |  | 0.13 | 0.85 | 0.005 |
| Kitchen | 0.24 | 0.33 | 0.27 |  | 0.16 | 0.32 | 0.45 |
| Bathroom | 0.16 | 0.12 | 0.64 |  | 0.20 | 0.74 | 0.11 |
| Bedroom | 0.20 | 0.37 | 0.41 |  | 0.13 | 0.26 | 0.67 |
| Living room | 0.09 | -0.04 | 0.87 |  | 0.10 | -0.03 | 0.92 |
| Public corridor in building | 0.24 | 1.93 | 0.0002 |  | 0.07 | 1.52 | 0.006 |
| Wash basin | 0.31 | 0.23 | 0.32 |  | 0.33 | 0.74 | 0.05 |
| **PR_t_ = PR_0_ – K × T** | **T = days after patient diagnosed** | | |  | **T = days after patient transported** | | |
|  | **PR_0_** | **K** | ***p for K*** |  | **PR_0_** | **K** | ***p for K*** |
| Residence | 0.11 | 0.02 | 0.01 |  | 0.09 | 0.02 | 0.03 |
| Kitchen | 0.17 | 0.02 | 0.40 |  | 0.17 | 0.05 | 0.32 |
| Bathroom | 0.15 | 0.01 | 0.59 |  | 0.20 | 0.09 | 0.07 |
| Bedroom | 0.16 | 0.03 | 0.57 |  | 0.13 | 0.03 | 0.65 |
| Living room | 0.09 | -0.004 | 0.88 |  | 0.10 | -0.005 | 0.88 |
| Public corridor in building | 0.03 | 0.003 | 0.44 |  | 0.03 | 0.005 | 0.32 |
| Wash basin | 0.27 | 0.03 | 0.25 |  | 0.31 | 0.14 | 0.02 |

Table S5 Correlation coefficients between positive rates of SARS-CoV-2 (PR_CoV_) in the public corridor and the cumulative numbers of newly diagnosed patients in the same building from the day of patient transport.

| **Time** | **Correlation coefficient** |
| --- | --- |
| 7^th^ day before patient transport | 0.14 |
| 6^th^ day before patient transport | 0.14 |
| 5^th^ day before patient transport | 0.14 |
| 4^th^ day before patient transport | 0.14 |
| 3^rd^ day before patient transport | 0.15 |
| 2^nd^ day before patient transport | 0.19 |
| 1^st^ day before patient transport | 0.21 |
| The day of patient transport | 0.38** |
| 1^st^ day after patient transport | 0.34** |
| 2^nd^ day after patient transport | 0.28* |
| 3^rd^ day after patient transport | 0.26* |
| 4^th^ day after patient transport | 0.19 |
| 5^th^ day after patient transport | 0.15 |
| 6^th^ day after patient transport | 0.12 |
| 7^th^ day after patient transport | 0.12 |

**p* < 0.05, ***p* < 0.01, ****p* < 0.001.

Figure S1 Heatmaps of positive rates of SARS-CoV-2 (PR_CoV_) in residences of A) single patient and B) multiple patients.
